# New Labyrinth Microfluidic Device Detects Circulating Tumor Cells Expressing Cancer Stem Cell Marker and Circulating Tumor Microemboli in Hepatocellular Carcinoma

**DOI:** 10.1038/s41598-019-54960-y

**Published:** 2019-12-09

**Authors:** Shanshan Wan, Tae Hyun Kim, Kaylee J. Smith, Ryan Delaney, G-Su Park, Hui Guo, Eric Lin, Thomas Plegue, Ning Kuo, John Steffes, Christopher Leu, Diane M. Simeone, Nataliya Razimulava, Neehar D. Parikh, Sunitha Nagrath, Theodore H. Welling

**Affiliations:** 1 Perlmutter Cancer Center and Department of Surgery, NYU Langone Health, New York, NY USA; 2 Department of Chemical Engineering, University of Michigan, Ann Arbor, MI USA; 3 Electrical Engineering and Computer Science, University of Michigan, Ann Arbor, MI USA; 4 Biointerfaces Inst., University of Michigan, Ann Arbor, MI USA; 5 Department of Chemistry, University of Michigan, Ann Arbor, MI USA; 6 School of Medicine, University of Michigan, Ann Arbor, MI USA; 7 Department of Internal Medicine, University of Michigan, Ann Arbor, MI USA; 8 Rogel Cancer Center, University of Michigan, Ann Arbor, MI USA; 9 Department of Breast and Thyroid Surgery, Union Hospital, Tongji Medical College, Huazhong University of Science and Technology, Wuhan, China

**Keywords:** Hepatocellular carcinoma, Translational research

## Abstract

Hepatocellular Carcinoma (HCC) is one of the most lethal cancers with a high mortality and recurrence rate. Circulating tumor cell (CTC) detection offers various opportunities to advance early detection and monitoring of HCC tumors which is crucial for improving patient outcome. We developed and optimized a novel Labyrinth microfluidic device to efficiently isolate CTCs from peripheral blood of HCC patients. CTCs were identified in 88.1% of the HCC patients over different tumor stages. The CTC positivity rate was significantly higher in patients with more advanced HCC stages. In addition, 71.4% of the HCC patients demonstrated CTCs positive for cancer stem cell marker, CD44, suggesting that the major population of CTCs could possess stemness properties to facilitate tumor cell survival and dissemination. Furthermore, 55% of the patients had the presence of circulating tumor microemboli (CTM) which also correlated with advanced HCC stage, indicating the association of CTM with tumor progression. Our results show effective CTC capture from HCC patients, presenting a new method for future noninvasive screening and surveillance strategies. Importantly, the detection of CTCs with stemness markers and CTM provides unique insights into the biology of CTCs and their mechanisms influencing metastasis, recurrence and therapeutic resistance.

## Introduction

The incidence of hepatocellular carcinoma (HCC) has doubled in the last few decades, having the fastest rising incidence among other solid malignancies in the US^1–3^. Over 50% of HCC cases are caused by chronic hepatitis B and C; while the recent sharp increase of liver cancer is due to the rise of alcoholic steatohepatitis and obesity linked non-alcoholic steatohepatitis (NASH)^4^. HCC has one of the highest mortality rates among solid organ cancers with a 5-year survival rate of only 15%^5^. Diagnosis at advanced stages occurs often and is associated with worse prognosis. Over two-thirds of HCC patients are diagnosed at an advanced stage, and these patients have a median survival of less than 1 year^6^. Only a subset of patients with early HCC stage are eligible for potential curative strategies such as resection, ablation, or liver transplant.

Current early detection strategies include abdominal ultrasound with or without AFP every 6 months, but they have inadequate sensitivity^7^. Thus, it is paramount to develop a more sensitive screening and surveillance tool for HCC. In addition, tumor recurrence and metastasis continues to be a large problem for HCC patients, with over 50% of patients developing recurrent HCC after primary resection, and with 15% of HCC patients developing extrahepatic metastasis^1,8^. Metastasis and relapse are often initiated by circulating tumor cells (CTCs) that penetrate the vasculature, disseminate through the bloodstream to other sites, and eventually form metastatic tumors^9,10^. The presence of CTCs and their number are a strong predictor of disease outcome in several cancer types^11^. Reliable detection and characterization of rare CTCs in HCC patients may facilitate early detection, provide additional prognostic information, and identify mechanisms of tumor progression and metastasis.

Although CTC detection is a promising diagnostic and monitoring technique, it remains challenging due to difficulty in sampling the extremely low concentrations of CTCs, with only 1–10 CTCs per mL in blood^10^. This has led to the development of many enrichment techniques. Currently, CellSearch^®^ is the only FDA-approved blood test for enumeration of CTCs in metastatic breast, colorectal, and prostate cancers; however, CTCs were only detected in 36% of metastatic cancer patients^12^. CellSearch^®^ isolates cells based on their expression of epithelial cell adhesion molecule (EpCAM) on the cell surface^13^. While this technique can detect some CTCs, it fails to isolate CTCs that do not express EpCAM^14^, including cells that have undergone an epithelial to mesenchymal transition (EMT) which downregulates EpCAM and promotes cell mobility^15^. Furthermore, HCC tumor cells are phenotypically highly heterogeneous. Only 35% of HCC cases were found to be positive for EpCAM, and even in “EpCAM positive” HCC, many still contained EpCAM negative tumor cells^16^. EpCAM-based methods would therefore be limited in sensitivity to detect HCC CTCs^17^. Thus, alternative enrichment methods and quantification markers are needed for reliable detection of HCC CTCs.

In an effort to increase CTC capture, microfluidic technologies have evolved since the first immunoaffinity-based CTC-Chip^18^. However, most affinity-based approaches rely on EpCAM due to the lack of known markers that could differentiate CTCs from normal blood cells. Recently, several label-free devices were developed utilizing the size-based differential focusing of CTCs^19^. One such device is the Labyrinth which utilizes inertial forces to focus CTCs and white blood cells (WBCs) into separate streamlines. This device has been used to isolate CTCs and characterize them from peripheral blood of breast and pancreatic cancer patients^20^.

In this study, we designed and optimized a new Labyrinth device to specifically capture CTCs from peripheral blood of HCC patients. To identify HCC CTCs, we combined three clinical grade antibodies against HCC markers widely used in diagnostic pathology: Glypican 3 (GPC3), Glutamine Synthase (GS), and Hep Par-1. In addition, HCC CTCs were counterstained using cancer stem cell (CSC) marker, CD44, which have been found to be tightly linked with tumor initiation, recurrence, and metastasis through their self-renewal and survival advantages^21,22^. Finally, circulating tumor microemboli (CTM), which could possess higher metastatic potential and resistance to apoptosis than single CTCs were quantified and correlated to HCC stages. These results show the capability to use our technology to further biological analysis of these rare cells in order to better understand the roles of CTCs and CTM in metastasis, therapeutic resistance, and relapse.

## Materials and Methods

### Labyrinth device fabrication

The Labyrinth chip was fabricated using soft lithographic techniques as shown previously^20^. In brief, a silicon master mold was created by spin coating a negative photoresist (SU-8 100, MicroChem) on a 4-inch wafer to a height of 110 μm and patterned subsequently using conventional photolithography. The height was measured using a surface profilometer (Veeco Dektak 6M). A 10:1 mixture of polydimethylsiloxane (PDMS) pre-polymer and curing agent was degassed, poured onto the mold, and baked overnight at 65 °C. The PDMS structure was peeled off from the mold, cut into a desired shape, and through-holes were created at both ends of the microchannel using a biopsy punch to form the inlet and outlets. Finally, the PDMS fluidic channel was bonded to a glass slide via plasma treatment and connected to an external tube set. All devices were individually inspected under the microscope and tested for leakage before use.

### Cell culture and labeling

Three human HCC cell lines (Hep G2, Hep 3B, PLC/PRF/5) were purchased from the American Type Culture Collection (ATCC, LGC Standards). These cells were cultured at 37 °C with 5% CO_2_ and maintained by regular passage in complete media consisting of RPMI 1640 Medium with 10% fetal bovine serum (FBS) and 1% penicillin-streptomycin solution (GIBCO®, Life Technology). When cells reached a confluency of 70–80%, they were collected and fluorescently labeled with green cell tracker dye (Invitrogen, CellTracker Green CMFDA, C7025) according to manufacturer instructions. HCC cells from these cell lines were diluted into non-HCC subject blood samples or PBS buffer solution for use in Labyrinth cell recovery experiments.

### Blood sample collection and processing

All research and experimental protocols were approved by an Institutional Review Board (IRB) at the University of Michigan. All study and methods were performed in accordance with the ethical regulations under an IRB-approved protocol at the University of Michigan. Informed consent was obtained from all participants. Blood samples from a pilot cohort of 12 HCC patients and 4 non-HCC patients were utilized for marker validation. Blood samples from 42 HCC patients were used to study HCC CTCs with relevant clinical information. Staging was assessed according to radiographic assisted RECIST v1.1 criteria. The TNM staging of HCC used in our study is based on the American Joint Committee on Cancer (AJCC) tumor/node/metastasis (TNM) classification system. T refers to primary tumor characteristics; N refers to regional lymph node involvement; M refers to distant metastasis status. HCC TNM stages I, II, and III are different T stages without lymph node metastasis (N0) and distant metastasis (M0). TNM Stage I: T1 (solitary tumor ≤2 cm or >2 cm without vascular invasion); TNM stage II: T2 (solitary tumor >2 cm with vascular invasion or multiple tumors, none >5 cm); TNM stage III: T3 (multiple tumors, at least one of which is >5 cm) and T4 (tumor involves a major branch of the portal vein or hepatic vein or tumor directly invades adjacent organs other than the gallbladder or tumor perforates the visceral peritoneum); TNM stage IV: any T stages with lymph node metastasis (N1) and/or distant metastasis (M1). A total of 10 mL of whole blood was drawn from patients diagnosed with HCC and non-HCC (healthy/control) subjects. All specimens were collected in EDTA tubes and processed within 4 hours of blood draw. Prior to sample injection, the Labyrinth was conditioned with 1% pluronic acid solution for 10 minutes to prevent cell adhesion on the channel surface and washed with PBS. Concurrently, blood samples were mixed with 6% dextran and kept at room temperature for 1 hour to remove red blood cells (RBCs) using density gradient separation. The supernatant was collected and diluted with PBS to a final volume of 50 mL. Samples were then processed through the Labyrinth twice at a target flow rate using a syringe pump (Harvard Apparatus) for CTC separation. CTCs isolated from the device were directly collected into a cytospin funnel to minimize cell loss during the process and centrifuged in Cytospin 4 cytocentrifuge (Thermo Scientific, Massachusetts) on a cytoslide at a speed of 800 rpm for 10 minutes. Finally, cells were fixed with 4% paraformaldehyde (Thermo Scientific, Massachusetts) for 15 minutes followed by a PBS wash and stored at 4 °C before further characterization.

### Immunofluorescence (IF) analysis

Processed blood samples were permeabilized with 0.1% Triton-X and blocked with 10% donkey serum for 30 minutes. Immunofluorescence (IF) staining was conducted using primary antibodies against a combination of markers including the following: Glypican 3 (Biocare Medical, California), Glutamine Synthetase (Biocare Medical, California), HepPar-1 (Dako, Denmark), CD44 (Atlas, Sweden), and CD45 (BD, New Jersey). Secondary antibodies conjugated with Alexa Fluor 488, 546, or 647 (Invitrogen) were diluted in 1% BSA and used for detection. The cytoslides were mounted with Prolong Gold Antifade Mountant with 4′, 6-diamidino-2-phenylindole (DAPI) (Thermo Scientific, Massachusetts) to counterstain the cell nuclei and stored at 4 °C until imaging. Slides were observed under 20x magnification using the inverted immunofluorescence microscope (Ti Eclipse, Nikon, New York) with an automated motor stage. Images were reviewed and counted manually.

### Statistical analysis

Data are presented as mean values ± standard error of the mean or as percent where appropriate. Difference in CTC counts between patients groups were evaluated using Mann-Whitney test. Differences in categorical variables such as percent positive CTCs were evaluated using Chi-square test. Significance was assigned for p-value less than 0.05. Statistical analyses were performed using GraphPad Prism software version 7.

## Results

### Labyrinth optimization for HCC CTC isolation

**Figure 1:**
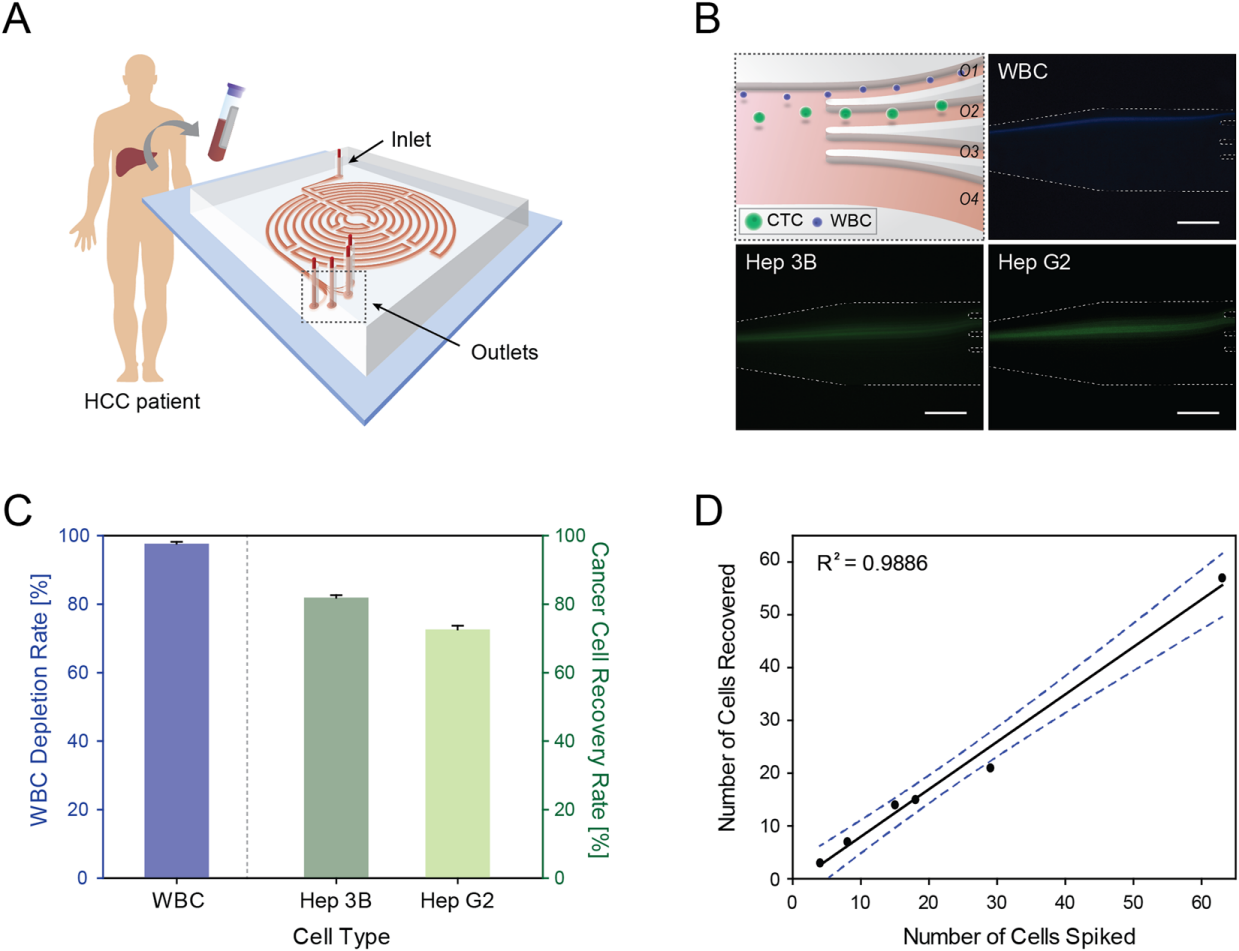
Characterization of the Labyrinth device with HCC cell lines. (**A**) CTC isolation workflow using the Labyrinth device. Red blood cells (RBCs) were removed from whole blood, sampled from HCC patients using density gradient separation prior to device injection. (**B**) CTC separation from white blood cells (WBCs) by differential inertial focusing and collection. Fluorescent microscope image of differentially focused cell streaks by inertial focusing and migration for cell separation. CTCs were collected through outlet 2 (O2). Scale bar represents 100 μm. (**C**) HCC cell line recovery and WBC depletion rate of the Labyrinth. Labyrinth operated at a constant flow rate of 2000 μl/min. Error bars represent the standard deviation of replicates. (**D**) CTC isolation performance. Small aliquots of HCC cells (5–100 Hep 3B cells) were spiked into whole non-HCC subject (control) blood and subjected to Labyrinth recovery.

The design of the Labyrinth and procedure to isolate CTCs from HCC patients are illustrated in Fig. 1A. The sharp corners in our Labyrinth device improved the focusing behavior of small WBCs by generating a strong instant Dean force that allows WBCs to migrate faster to their equilibrium position. The path of these corners was designed to be short enough, to not disturb the focusing streams. Prior to applying the Labyrinth to patient samples, the device was tested and optimized using Hep G2 and Hep 3B HCC cell lines. Since the Labyrinth utilizes a size-based differential focusing of cells generated by the balance between the inertial lift and Dean forces^20,23^, the diameter of each cell type was measured. Expectedly, both HCC cell lines were larger in diameter measuring 16.62 ± 2.84 μm and 14.49 ± 2.12 μm for Hep 3B and Hep G2, respectively, compared to WBCs (Supplementary Fig. 1A, n = 1000 per cell type).

Compared to our previous study^20^, the height of the Labyrinth was modified and increased to 110 μm. The extended height increased the Dean force effect, allowing the cells to equilibrate at similar focusing positions at lower flow velocities^23^. This reduced the overall shear stress acting on the cell surfaces during CTC separation. HCC cell lines were pre-labeled with CellTracker Green CMFDA dye while WBCs extracted from healthy donor blood were stained with DAPI. From a flow rate of 1800 μL/min, all cells started to focus and form tight streamlines by size. The focused cell streak of WBCs was located near the inner curvature of the channel wall whereas the larger HCC cell lines were positioned near the channel center due to the dominant lift forces. The difference in the cell streamlines formed along the channel allowed them to be collected into individual outlets for separation.

To determine the optimal flow rate for efficient CTC separation, a cell mixture of HCC cells and WBCs were diluted into buffer (5 × 10^6^ cells/mL) and injected into the Labyrinth at varying flow rates of 1800–2600 µL/min (Supplementary Fig. 1C–E). Cells were then collected from outlet O1 and O2 for WBCs and HCC cell lines respectively, to determine the overall recovery rates (Fig. 1B). Outlet O3 and O4 were included to simply reduce the sample fluid volume after cell separation and collection. Possible HCC cell clusters were expected to be collected by O2 from the specific outlet design. The maximum potential CTC recovery rate, using HCC cell lines, were 85.96 ± 0.55% and 83.07 ± 1.26% for Hep 3B and Hep G2 cells, respectively, at a flow rate of 1800 μL/min (*n* = 3). However, to further improve the purity of CTC collection, a higher flow rate of 2000 μL/min was determined to be optimal. At this flow rate, the cut-off cell diameter entering the collection outlet O1 and O2 was approximately 13 μm. The overall CTC recovery rate of 81.89 ± 0.73% and 72.57 ± 1.15% was achieved for Hep 3B and Hep G2 cells, respectively, with a WBC depletion rate of 97.66 ± 0.53% (Fig. 1C). The difference in capture rate for the two HCC cell lines was due to the difference in average cell diameter. Importantly, considering only those cells with a cell size above the cut-off cell diameter entering the collection outlet O2 (Supplementary Fig. 1B**)**, the CTC recovery rate for HCC cell lines became 91.17% and 95.69% for Hep 3B and Hep G2 cells respectively.

Above this flow rate, the cell isolation efficiency decreased as the streamlines of both HCC cells steadily migrated towards the inner channel wall closer to that of WBCs by an increasing Dean Force effect. Across all flow rates tested, more than 90% of the cells were intact and viable after running through the Labyrinth (data not shown). Finally, to mimic the presence and rarity of HCC CTCs in patient blood, a small number of Hep 3B cells (5–100 cells) were spiked into 5 mL of non-HCC subject (healthy/control) blood samples, treated with dextran to remove RBCs, and HCC cells isolated at a flow rate of 2000 μL/min using Labyrinth. A linear capture rate was observed along this range of small number of HCCs cell spiked into whole blood (R^2^ = 0.99) (Fig. 1D).

### Detection and characterization of CTCs in HCC patients

The total number of CTCs in blood samples from 42 HCC patients were evaluated along with 5 non-HCC healthy subjects as controls. The baseline characteristics of the HCC cohort are summarized in Table 1. The median age of HCC patients was 66, ranging from 54 to 88 years of age. Hepatitis C was the most common liver disease etiology (n = 24, 57.1%) followed by NASH (n = 10, 23.8%), and alcohol-related disease (n = 4, 9.5%). No underlying liver disease was noted in 4 patients (9.5%). 41 patients had newly diagnosed and untreated HCC at the time of blood collection while one patient had undergone cryoablation recently and had no detection of HCC radiographically. According to AJCC TNM staging, 26 patients (61.9%) were stage II or above. Multiple tumor nodules were noted in 19 patients (45.2%) and radiographically evident macrovascular invasion was observed in 10 patients (23.8%).

**Table 1:** Patient clinical characteristics.

Clinical Characteristics		*n (%)*
**Age (y)**		
Median (66); Range (54–88)		
AFP (ng/mL)	≤20	*22 (53.66%)*
Median (17); Range (1–162650)	>20	*19 (46.34%)*
Tumor size (cm)	≤5	*22 (52.38%)*
Median (4.6); Range (0–10.8)	>5	*19 (45.24%)*
Tumor number	Single	*22 (52.38%)*
	Multiple	*13 (30.95%)*
TNM stage	T0	1 (2.4%)
	I	15 (35.7%)
	II	*8 (19%)*
	III	*12 (28.6%)*
	IV	6 (14.3%)
BCLC stage	0	3 (7.1%)
	A	11 (26.2%)
	B	15 (35.7)
	C	13 (30.95)
Cirrhosis	No	*7 (16.67%)*
	Yes	*35 (83.33%)*
Macrovascular invasion	No	*32 (76.19%)*
	Yes	*10 (23.81%)*
Etiology	HCV	*24 (57.14%)*
	HBV	*0 (0%)*
	ETOH	*4 (9.52%)*
	NASH	*10 (23.81%)*
	None	*4 (9.52%)*

HCC tumor cells are highly heterogeneous. To detect a wide range of HCC CTCs after collection from the Labyrinth device, three characteristic HCC markers including GPC3, GS, and Hep Par-1 were evaluated using IF analysis. Three HCC cell lines were used to validate and optimize the IF staining procedure (Supplementary Fig. 2). All three cell lines showed positive staining for GPC3 and GS. Hep G2 and Hep 3B showed negative staining for Hep Par-1, whereas PLC/PRF/5 showed positive staining for Hep Par-1. WBCs from healthy donors were used as negative controls to confirm the specificity of the antibodies. For further marker validation, we compared the detection rate for HCC CTCs using three HCC markers verses EpCAM in a pilot cohort of 12 HCC patients and 4 cholangiocarcinoma (CCA) patients. Labyrinth-enriched CTCs were subjected to IF staining for either combined three HCC markers or EpCAM. 3 out of 4 samples from non-HCC patients showed EpCAM positive cells, while all 4 of the non-HCC samples were negative for HCC markers, showing better specificity of HCC markers than EpCAM for identifying HCC CTCs (Supplementary Fig. 3C**)**. For the samples from HCC patients, 11 out of 12 (91.7%) had HCC markers-positive CTCs, one showed neither HCC makers nor EpCAM positivity (Supplementary Fig. 3C**)**. Among the 11 HCC samples with HCC markers-positive CTCs, 8 out of 11 (72.7%) also showed EpCAM-positive CTCs, while 3 out of 11 (27.3%) were EpCAM-negative (Supplementary Fig. 3C**)**. CTC positivity rate was improved in HCC patients by using three HCC markers (91.7%) compared to EpCAM (66.7%) (Supplementary Fig. 3D**)**. Therefore, combination of three HCC markers was chosen to increase the detection rate of CTCs in a study cohort of 42 HCC patients.

**Figure 2:**
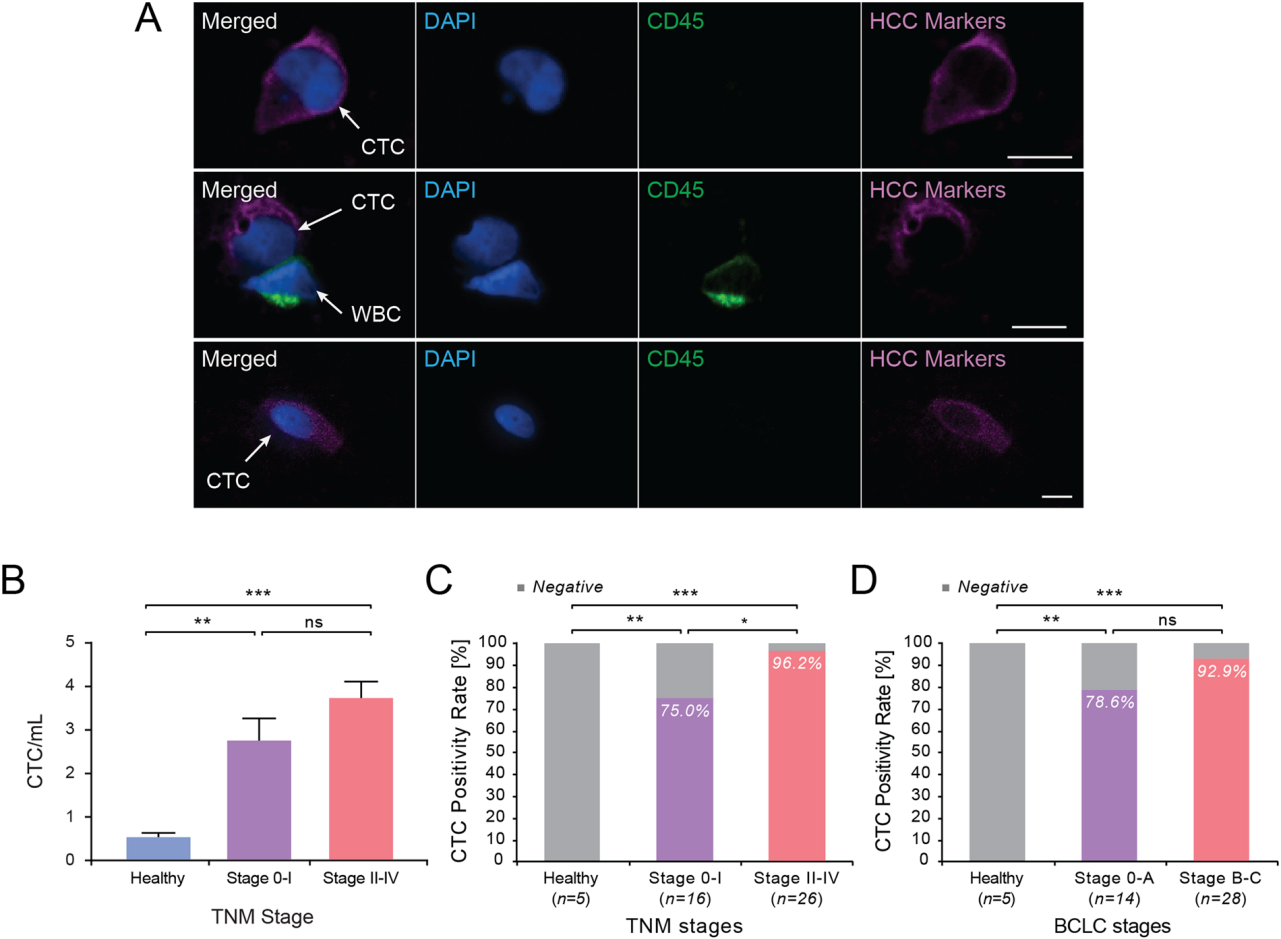
Identification and quantification of CTCs isolated from HCC patients. (**A**) Representative image of CTCs captured by the Labyrinth and stained using antibodies against Gly3, GS, Hep Par-1, CD45, and DAPI. Scale bar represents 10 μm. (**B**) Number of CTCs/mL in HCC patients grouped by TNM stage. **TNM stage 0-I vs non-HCC: p = 0.0019; ***stage II-IV vs non-HCC: p = 0.0001; stage 0-I vs II-IV, p = 0.1258 (ns). (**C**) CTC positivity rate in TNM stage. **non-HCC vs Stage 0-I: p = 0.0031; ***non-HCC vs Stage II-IV: p < 0.0001; *Stage 0-I vs II-IV: p = 0.0398. (**D**) CTC positivity rate in BCLC stage. **non-HCC vs Stage 0-A: p = 0.0048; ***non-HCC vs Stage B-C: p < 0.0001; Stage 0-A vs Stage B-C: p = 0.3126 (ns).

Using IF analysis of Labyrinth-processed blood samples, CTCs were identified as CD45 negative and HCC marker positive cells with intact nuclei stained by DAPI (Fig. 2A). The total number of CTCs detected from HCC patients ranged from 0.4 to 8.7 CTCs/mL (mean = 3.36 ± 0.31). A significantly higher number of CTCs was observed from HCC patients (mean = 3.36 ± 0.31 CTC/ml, n = 42) compared to that from healthy subjects (mean = 0.53 ± 0.1 CTC/ml, n = 5) (p = 0.0001). Patients with more advanced TNM stages (TNM II-IV) (3.73 CTCs/mL, n = 26) had higher number of CTCs compared to early stage HCC patients (TNM 0-I) (2.76 CTCs/mL, n = 16), however this did not reach statistical significance (p = 0.1258) (Fig. 2B). Using ≥1 CTC/mL as a cut-off for CTC positivity based on controls, 37 out of 42 HCC patients (88.1%) were positive for CTCs, whereas all 5 non-HCC (healthy/control) subjects were negative for CTCs. Using this criterion, the rate of CTC detection correlated in HCC patients along TNM stages, with 96.2% positivity in more advanced TNM stage (TNM II-IV) and 75% positivity in early TNM stage (TNM 0-I) (p = 0.0398) (Fig. 2C). Patients also had a higher rate of CTC detection at more advanced BCLC stages (BCLC B-C), but this was not statistically significant (p = 0.3126) (Fig. 2D).

Notably, 100% of patients with macrovascular invasion (n = 10) were positive for CTCs, whereas 84.4% of patients without macrovascular invasion (n = 32) had detectable CTCs, suggesting a link between CTC and invasive tumor. Due to the limited number of patients with macrovascular invasion, the data did not reach statistical significance (p = 0.1829) (Supplementary Fig. 4A). Other HCC prognostic factors did not clearly predict a higher CTC detection rate. Neither CTC positivity rate nor the number of CTCs showed a significant difference between high serum AFP versus low serum AFP groups using either 20 ng/mL or 100 ng/mL AFP as a cut-off value (Supplementary Fig. 4B). Likewise, there was no association between CTC positivity rate and HCC tumor size for tumors greater or less than 5 cm (Supplementary Fig. 4C). In summary, our data indicate that CTC positivity rate is significantly associated with HCC TNM stages and potentially linked with macrovascular invasion.

### Detection of CD44^+^ CTCs in HCC patients

**Figure 3:**
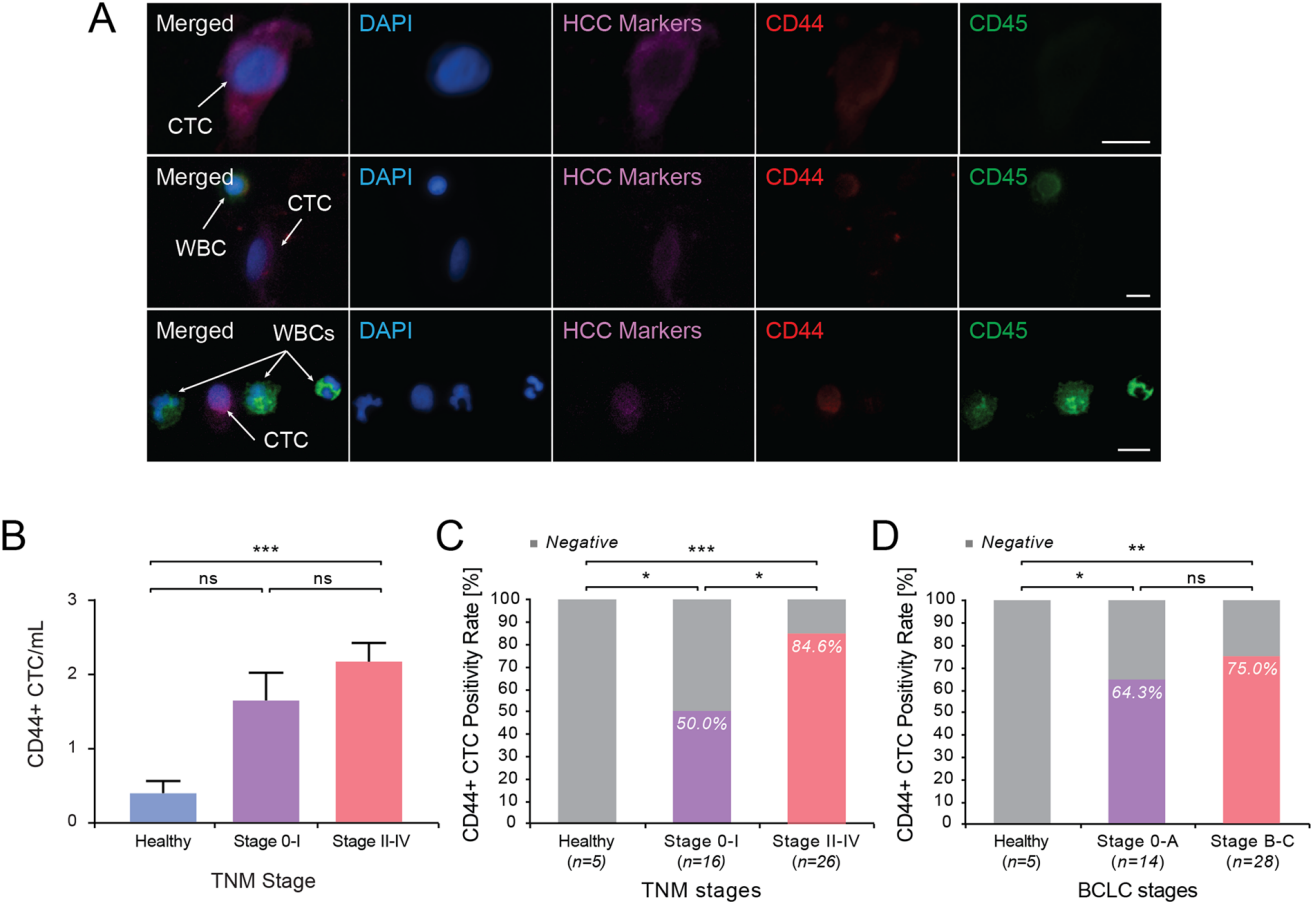
Identification and quantification of CD44 positive CTCs in HCC patients. (**A**) Representative image of CTCs and WBCs additionally stained with antibody against CD44. Scale bar represents 10 μm. (**B**) Number of CD44^+^ CTCs/mL in HCC patients grouped by TNM stage. TNM stage 0-I vs non-HCC: p = 0.1130 (ns); ***stage II-IV vs non-HCC: p = 0.0001; stage 0-I vs II-IV, p = 0.0765 (ns). (**C**) CD44^+^ CTC positivity rate in TNM stage. *non-HCC vs Stage 0-I: p = 0.0445; *** non-HCC vs Stage II-IV: p = 0.0001; *Stage 0-I vs II-IV: p = 0.0159. (**D**) CD44^+^ CTC positivity rate in BCLC stage. *non-HCC vs Stage 0-A: p = 0.0325; **non-HCC vs Stage B-C: p = 0.0033; Stage 0-A vs Stage B-C: p = 0.4913 (ns).

Cancer stem cells (CSCs) possess the ability to self-renew and sustain the tumor, as well as give rise to metastasis and recurrence. CD44 is a widely used marker for cancer stem cells in solid tumors including HCC. To examine whether HCC CTCs had a cancer stemness phenotype, three-color IF staining was performed for all subjects using an additional anti-CD44 antibody (Fig. 3A). Among HCC marker positive CTCs, CD44^+^ CTCs were detected in all stages of HCC. The mean percentage of CD44^+^ CTCs from all HCC patients was 76.2 ± 2.8%, ranging from 37% to 100%. The percentage of CD44^+^ CTCs was not different between groups with more advanced TNM stage (77.1% ± 4.9%) versus early TNM stage (74.6 ± 4%). Hence, CD44^+^ CTCs were a major proportion of HCC CTCs across all stages of HCC. The mean number of CD44^+^ CTCs was higher in patients with more advanced TNM stage (2.17 CTCs/mL, n = 26) than those with early TNM stage (1.65 CTCs/mL, n = 16) (p = 0.0765) (Fig. 3B), though the p value is slightly above 0.05. Using ≥1 CD44^+^ CTC/mL as a cut-off for positivity, the positivity rate of CD44^+^ CTC was significantly higher in HCC patients with more advanced TNM stages (84.6%, n = 26), compared with those in early TNM stages (50%, n = 16) (p = 0.0159) (Fig. 3C). CD44^+^ CTC positivity rate was significantly higher in HCC patients than in non-HCC, healthy control subjects. When grouping patients by BCLC stages, no significant difference was observed between patients with Stage 0-A (64.3%, n = 14) and Stage B-C (75%, n = 28) (Fig. 3D). CD44^+^ CTC positivity rate trended higher in patients with macrovascular invasion (9 out of 10, 90%) than in patients without macrovascular invasion (21 out of 32, 65.6%), though it did not reach statistical significance (p = 0.1324) (Supplementary Fig. 5A). CD44^+^ CTC positivity rate showed no significant difference between high serum AFP patients and low serum AFP groups using either 20 ng/mL or 100 ng/mL as a cut-off. There was no difference in CD44^+^ CTC positivity between smaller versus larger (>5 cm) HCC tumors (Supplementary Fig. 5B,C).

### Detection of circulating tumor microemboli in HCC patients

**Figure 4:**
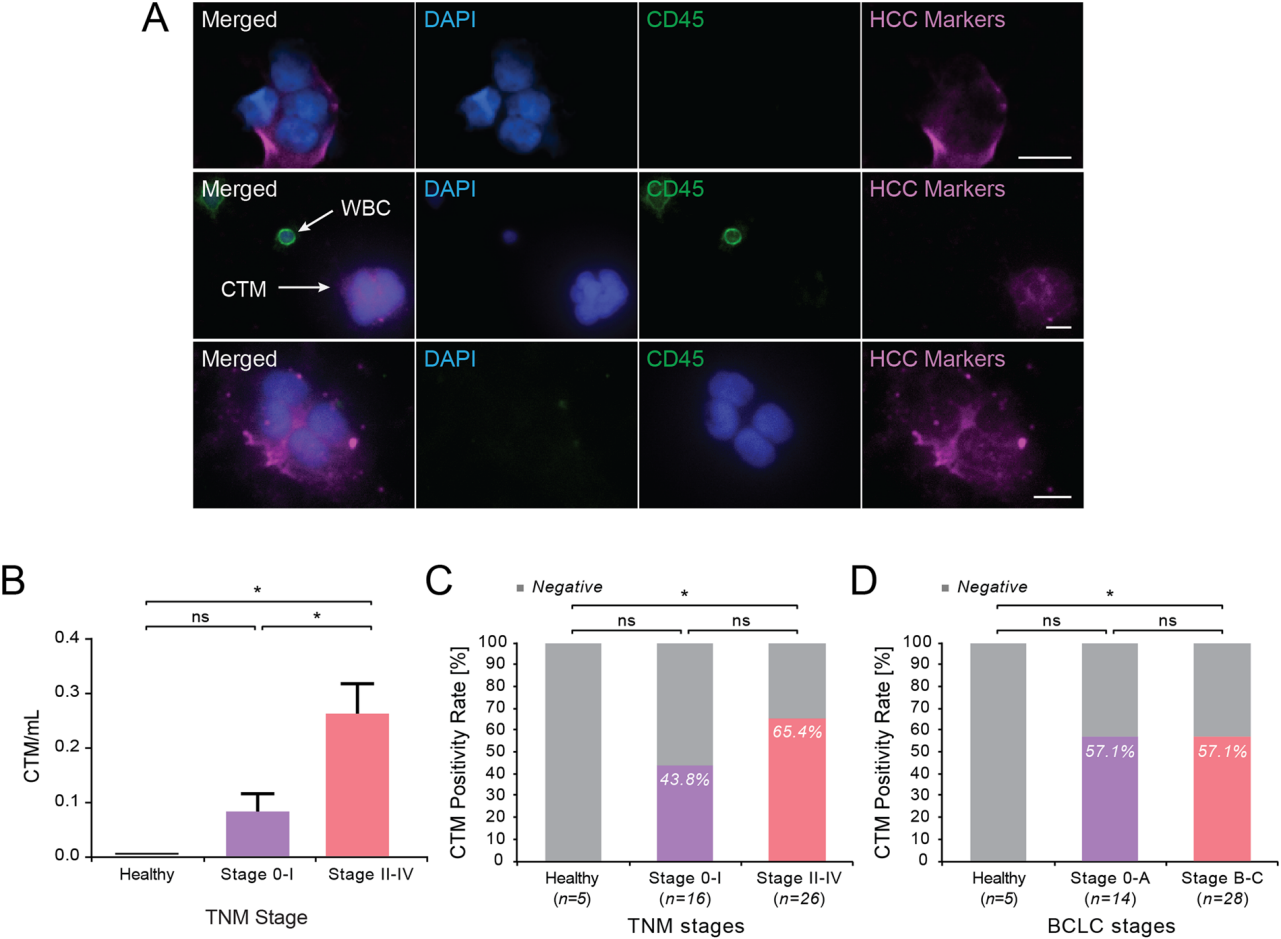
Identification and quantification of circulating tumor microemboli (CTM) in HCC patients. (**A**) Representative image of CTM and WBCs stained with antibodies against HCC markers and CD45. Scale bar represents 10 μm. (**B**) Number of CTM/mL in HCC patients grouped by TNM stage. TNM stage 0-I vs non-HCC: p = 0.2415 (ns); *stage II-IV vs non-HCC: p = 0.0195; *stage 0-I vs II-IV, p = 0.0172. (**C**) CTM positivity rate in TNM stages. Non-HCC vs Stage 0-I: p = 0.1235 (ns); *non-HCC vs Stage II-IV: p = 0.0118; Stage 0-I vs II-IV: p = 0.2097 (ns). (**D**) CTM positivity rate in BCLC stage. non-HCC vs Stage 0-A: p = 0.2621 (ns); *non-HCC vs Stage B-C: p = 0.0118; Stage 0-A vs Stage B-C: p = 0.1133 (ns).

Circulating tumor microemboli (CTM) or CTC clusters were also observed (Fig. 4A) among the CTCs isolated from HCC patients. Since CTM could possess higher metastatic potential^24^, we sought to quantify the significance of HCC CTM. CTM were observed in all stages of HCC (mean = 0.19 ± 0.04, n = 42) ranging from 0 to 1.29 CTM/mL, but were not observed in any non-HCC control subjects (n = 5) (Fig. 4B). The number of CTM was significantly higher in more advanced TNM stages (mean = 0.26 ± 0.06 n = 26), compared with early TNM stages (mean = 0.08 ± 0.03, n = 16) (p = 0.0172). Samples with at least one CTM detected were characterized as positive for CTM. The positivity rate for CTM trended higher in more advanced TNM stages (65.4%), compared with early TNM stage (37.5%), although this was not significant (p = 0.078); whereas a similar trend was not noted across BCLC stages (Fig. 4C,D). The detection rate of CTM trended higher in patients with macrovascular invasion (70%, n = 10) compared to patients without invasion (50%, n = 32), but this was not significant (p = 0.3049) (Supplementary Fig. 6A). CTM positivity rate did not show significant difference between low serum AFP and high serum AFP HCC patients using either 20 or 100 ng/mL AFP as cut off, nor between HCC patients of tumor size larger versus smaller than 5 cm diameter (Supplementary Fig. 6B,C).

## Discussion

HCC continues to be a cancer with rising incidence, high mortality, and recurrence rate. It can be cured if detected at an early stage and has well-defined risk factors including cirrhosis, HBV, HCV, and NASH. Thus, the American Association for the Study of Liver Disease (AASLD) guidelines recommends surveillance for high-risk populations in order to detect HCC at an early stage. Abdominal ultrasound and peripheral blood AFP measurement are commonly used for HCC screening. However, not all patients benefit from this surveillance strategy, ultrasound has a sensitivity of approximately 60%; whereas AFP has an even lower sensitivity ranging from 25%-60%^7^. In addition, high recurrence rate of HCC after resection or ablation remains a significant challenge. Thus it is extremely critical to develop new technologies to efficiently screen and monitor high risk patients.

CTCs have emerged as a promising tool for assessing tumor burden, noninvasive monitoring of cancer progression, and response to therapy, as well as a better understanding of blood-borne metastasis^19^. CTCs are extremely rare events and difficult to detect and characterize. To isolate CTCs from HCC patients, several studies have utilized EpCAM to identify HCC CTCs. However, only 35% of HCC patient tissues are EpCAM positive, and even in the EpCAM positive HCC tumors, there is a high frequency of EpCAM negative HCC cells. In addition, the high plasticity of HCC cells due to epithelial to mesenchymal transition (EMT) leads to decreased sensitivity of CTC detection using platforms based on epithelial characteristics (EpCAM) of CTCs. Therefore, EpCAM-based platforms have largely reduced sensitivity for capturing HCC CTCs. Two studies using CellSearch® resulted in a low HCC CTC detection rate of 30.5% and 66.7% respectively^25–27^. Hence, alternative technology for CTC detection in HCC needs to be developed to overcome the limitations of current approaches.

To improve HCC CTC detection rate, identify a variety of HCC CTCs, and allow future downstream analysis capacity, label-free size-based microfluidic device is especially attractive. Size based CTC isolation devices include filtration and inertial methods^12^. However, filtration approaches suffer from pore clogging and high-pressure drop which may cause cell damage and possibly encounter CTC loss from forcing the cells to squeeze through the pores. This limits throughput and the amount of blood being processed. Alternatively, others have utilized inertial forces to isolate CTCs with high-throughput capacity using sinusoidal or spiral channel geometries. Our high-throughput label-free Labyrinth device has demonstrated successful enrichment of CTCs from patients with breast cancer and pancreatic cancer^20^. The Labyrinth offers unique features over previous inertial devices by incorporating 56 sharp corners that allows focusing of smaller cells which was difficult to achieve using previous technology.

Here, we optimized the label-free Labyrinth technology to capture heterogeneous populations of CTCs from peripheral blood samples of 42 patients with HCC (Supplementary Table 1**)**. To detect and quantify HCC CTCs isolated using the Labyrinth device, we incorporated an HCC panel containing three clinically established HCC markers, GPC3, Hep Par1, and GS to improve the detection rate. GPC3 is a protein that is abundantly expressed in fetal liver, lost in mature hepatocytes, but commonly expressed in HCC. It is a member of the glypican family of heparan sulfate proteoglycans attached to cell surface through glycosyl-phosphatidylinositol anchor. GPC3 has been found to be significantly upregulated in HCC tumor cells, with around 80% positivity rate in HCCs^28^. GPC3 expression has been observed more frequently in moderately and poorly differentiated HCC compared to well-differentiated HCC, and correlated with poor prognosis^29^. HepPar-1 is another clinical marker for HCC, with positivity rate ranging from 75% to 90% for all HCCs and less frequently observed in poorly differentiated HCCs (50%-60%)^30^. GS is overexpressed in HCC, with positive staining ranging from 45%-78% of HCC^31,32^. GS expression has been observed to be highly increased in advanced HCC and associated with shorter relapse-free survival, proposing a role of GS in promoting metastatic potential of HCC^33^. Collectively, greater than 80%, 70%, and 80% of HCC specimens have been found to be positive for GPC3, GS, and Hep Par1, respectively^34,35^. As hypothesized, the HCC CTC detection rate was improved by using the three HCC markers compared to EpCAM based identification method (Supplementary Fig. 3A–D, Table S1).

We detected CTCs in 88.1% of HCC patients showing at least 1 CTC/mL. CTCs were detected in 75% of patients with early stage HCC (TNM 0/I) and 96.2% with more advance stage HCC (TNM II-IV). We also detected CTCs from one stage 0 patient who had HCC treated by ablation and had no disease detected by imaging. CTCs from this stage 0 patient could be derived from the original HCC tumor and disseminated in the peripheral blood before the treatment or from newly developed HCC that had yet to be detected by imaging. Based on TNM stages, intermediate to advanced HCC patients in this cohort demonstrated higher CTC number, and all had more than one tumor nodule. Both evidences suggest that CTCs may be able to indicate tumor burden, progression, and aggressiveness. The CTC counts did not statistically correlate with BCLC stages. BCLC stage system is different from TNM stage system in that it includes other clinical components besides tumor burden. BCLC staging system specifically includes the severity of liver disease and physical performance status. This may explain why CTCs correlated with TNM stages (tumor burden), whereas BCLC stages reflect other clinical factors unrelated to tumor burden leading to less association with the number of CTCs.

Recurrence and metastasis are the two major causes of cancer related death. CSCs are cells that can self-renew, differentiate into other tumor cell phenotypes, and form new tumors^36^. CSCs have been tightly linked with recurrence and metastasis through their drug resistance and survival advantages^37^. CD44 is a common surface marker for CSCs in many cancers including HCC^22^. The observation that all patients with macrovascular invasion were positive for CTCs (n = 10), and the positivity rates of both CD44^+^ CTCs and CTM had trended higher in patients with invasion than those without invasion, suggested that CD44^+^ CTCs and CTM may be linked with higher invasive properties. Since only 10 patients in this cohort had macrovascular invasion, no statistical significance was achieved between patients with invasion and those without invasion. Larger study cohorts will be needed in the future to determine the link between invasion and CD44^+^ CTCs or CTM.

In conclusion, we developed a Labyrinth device coupled with three clinically validated markers to isolate and quantify CTCs from blood samples of patients with HCC. Our methodology markedly improved the capability to isolate heterogeneous HCC CTCs with high recovery rate and purity among all label-free technologies. The device was able to detect CTCs in the majority of patients with HCC, and the positivity rate of HCC CTCs was significantly correlated with HCC disease stages, demonstrating the clinical potential for early detection, treatment selection, and monitoring response to targeted therapies. We also provide evidence for the first time that HCC CTCs contain CD44^+^ cancer stem cell populations and CTM which were associated with tumor stage. The positivity rate of CD44^+^ CTCs was significantly higher in patients with more advanced TNM stages, suggesting that a subpopulation of HCC CTCs acquired stemness properties that provide survival advantages in peripheral blood. CTM or CTC clusters were also observed in all stages of HCC, possibly providing additional survival signal and metastasis potential^38,39^. In addition, our approach can be readily applied in future clinical studies by incorporating single-cell genomic and transcriptomic profiling of CTCs isolated from the Labyrinth device. This will provide opportunities to further study the mechanisms by which CTCs and CTMs contribute to the process of invasion, metastasis, and recurrence.

## Supplementary information

Supplementary Information

## Data Availability

The datasets generated during and/or analyzed during the current study are available from the corresponding author on reasonable request.
