# Supplementary material for: New Labyrinth Microfluidic Device Detects Circulating Tumor Cells Expressing Cancer Stem Cell Marker and Circulating Tumor Microemboli in Hepatocellular Carcinoma
Source: Sci Rep. 2019 Dec 9;9:18575. doi: 10.1038/s41598-019-54960-y (PMC6901480; doi:10.1038/s41598-019-54960-y)
Supplement: Supplementary file 1 — Supplementary Information [file 41598_2019_54960_MOESM1_ESM.docx]

**New Labyrinth Microfluidic Device Detects Circulating Tumor Cells
Expressing Cancer Stem Cell Marker and
Circulating Tumor Microemboli in Hepatocellular Carcinoma**

Shanshan Wan^†1^ ,Tae Hyun Kim^†2,3,4^, Kaylee Smith^2,4^, Ryan Delaney^2^, G-Su Park^5^, Hui Guo^6^, Eric Lin^2,4^, Thomas Plegue^2^, Ning Kuo^6^, John Steffes^6^, Christopher Leu^1^, Diane M. Simeone^1^, Nataliya Razimulava^7^, Neehar D. Parikh^7^, Sunitha Nagrath^*2,4,8^, and Theodore H. Welling^*1^

Perlmutter Cancer Center and Department of Surgery^1^, NYU Langone Health, New York, NY, USA

Department of Chemical Engineering^2^, Electrical Engineering and Computer Science^3^, Biointerfaces Inst.^4^, Department of Chemistry^5^, School of Medicine^6^, Department of Internal Medicine^7^, Rogel Cancer Center^8^, University of Michigan, Ann Arbor, MI, USA

^†^These authors contributed equally to this work.

*** Corresponding authors:**

- Theodore H. Welling^*^

Perlmutter Cancer Center and Department of Surgery, NYU Langone Health, 435 East 30^th^ Street, New York, NY 10016, USA, Phone: (212)-731-6110, [Theodore.Welling@nyumc.org](mailto:Theodore.Welling@nyumc.org)

- Sunitha Nagrath^*^

Department of Chemical Engineering, University of Michigan, 2800 Plymouth Road, Ann Arbor, MI 48109, USA, Phone: (734) 647-7985, [snagrath@umich.edu](mailto:snagrath@umich.edu)

**SUPPLEMENTARY INFORMATION**

**Figures and Tables**

**Supplemental Figure 1.** Flow rate optimization for Labyrinth based on size distribution of HCC cell lines.

**Supplemental Figure 2.** HCC marker characterization using HCC cell lines.

**Supplemental Figure 3.** Identification and quantification of HCC marker and EpCAM positive CTCs in HCC patients.

**Supplemental Figure 4.** CTC positivity rate and HCC prognostic factors.

**Supplemental Figure 5.** CD44 positive CTCs and HCC prognostic factors.

**Supplemental Figure 6.** CTM and HCC prognostic factors.

**Supplemental Table 1.** Comparison of HCC CTC studies and technologies used for isolation.

**
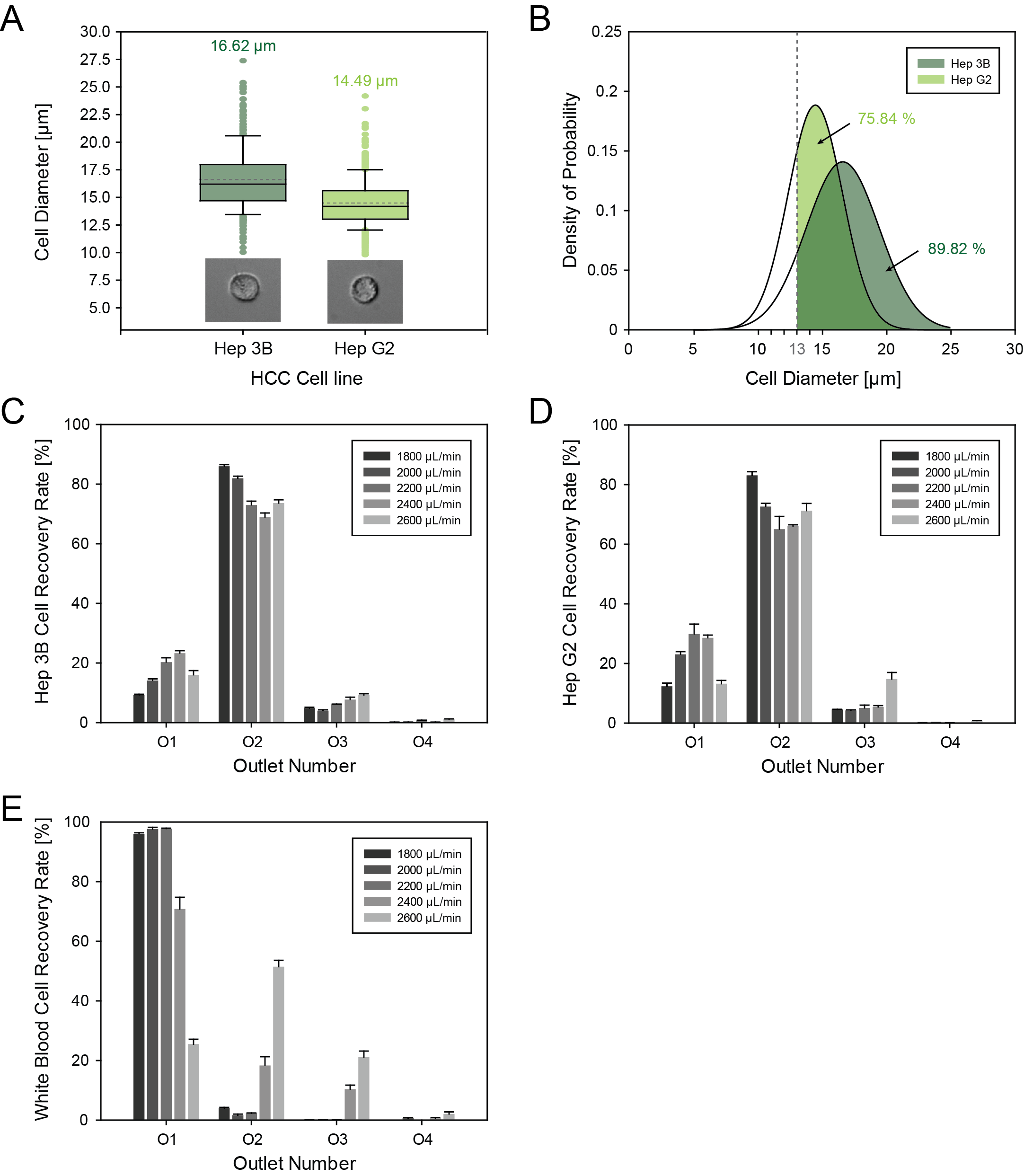
**

**Supplemental Figure 1. Flow rate optimization for Labyrinth based on size distribution of HCC cell lines.**

A. Cell size measurement and B. probability distribution of HCC cell lines. Cell collection rate of C. Hep 3B, D. Hep G2, and E. WBC from each outlet at varying indicated flow rates.


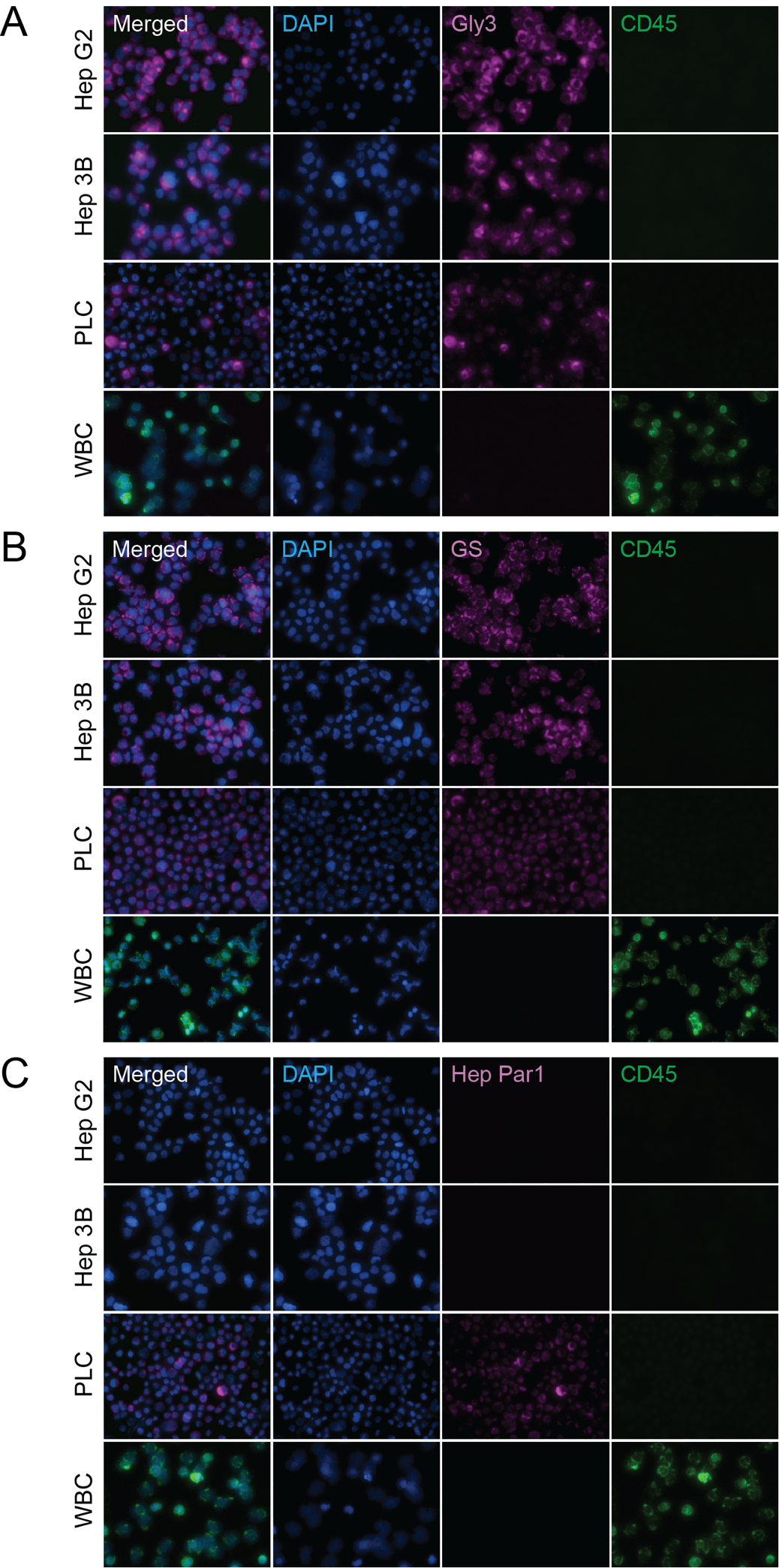


**Supplemental Figure 2. HCC marker characterization using HCC cell lines.** Fluorescent microscope image of three HCC cell lines stained with A. anti-Gly3, B. anti-GS, C. anti-Hep Par-1, and DAPI. WBCs extracted from non-HCC donor blood were used as a negative control. Anti-CD45 was counterstained to test the specificity of the immunofluorescent analysis.


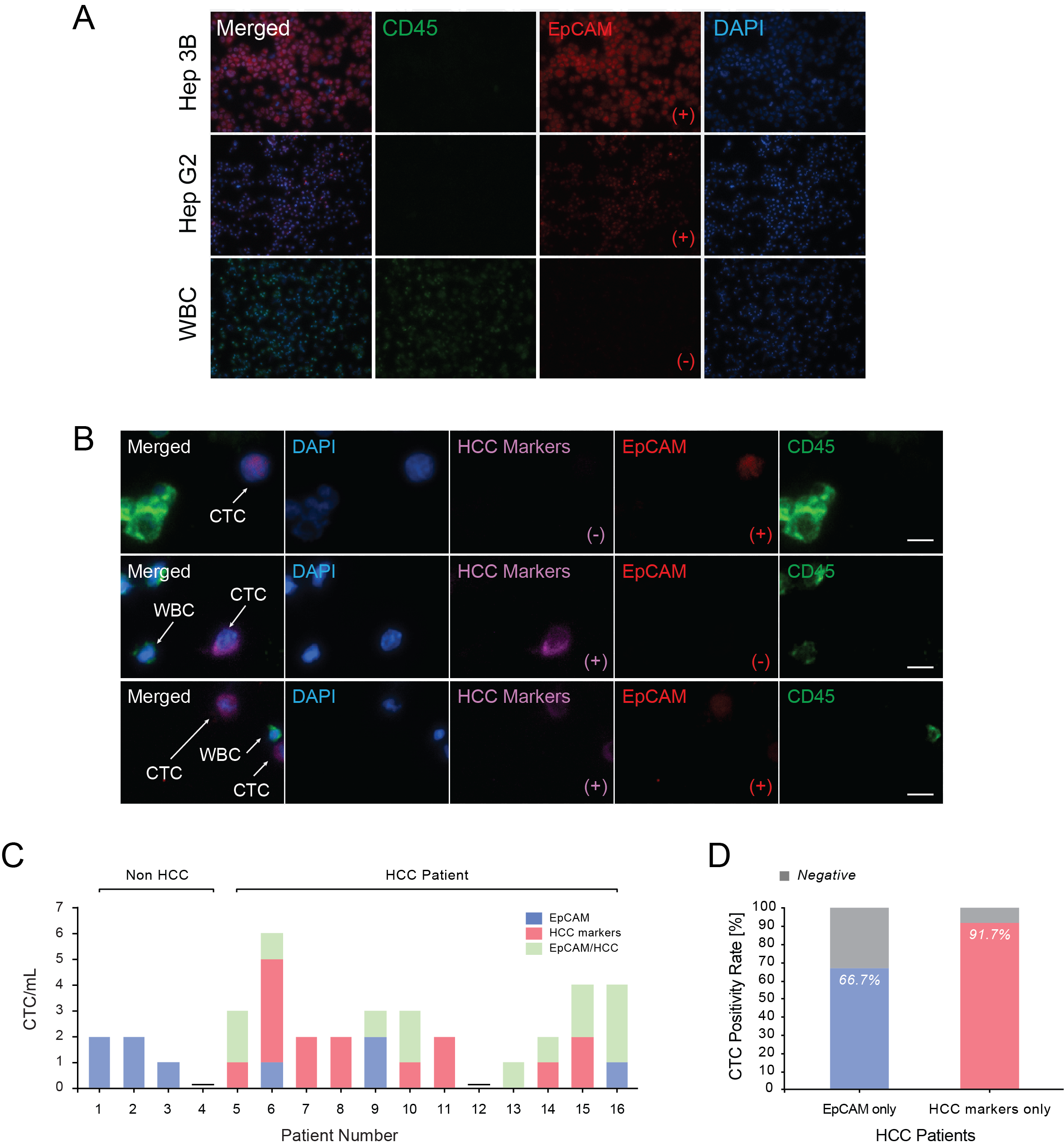


**Supplemental Figure 3. Identification and quantification of HCC marker and EpCAM positive CTCs in HCC patients.**

A. Fluorescent microscope image of HCC cell lines stained with EpCAM, DAPI and CD45. WBCs were tested as negative control. B. Representative image of CTCs stained using antibodies against three HCC markers (Gly3, GS, and Hep Par-1), EpCAM, CD45, and DAPI. Scale bar represents 10 μm. C. Number of CTCs/mL identified using HCC markers and EpCAM in HCC and non-HCC (Cholangiocarcinoma) patients. D. Comparison of CTC detection rate using EpCAM and HCC markers in HCC patients.


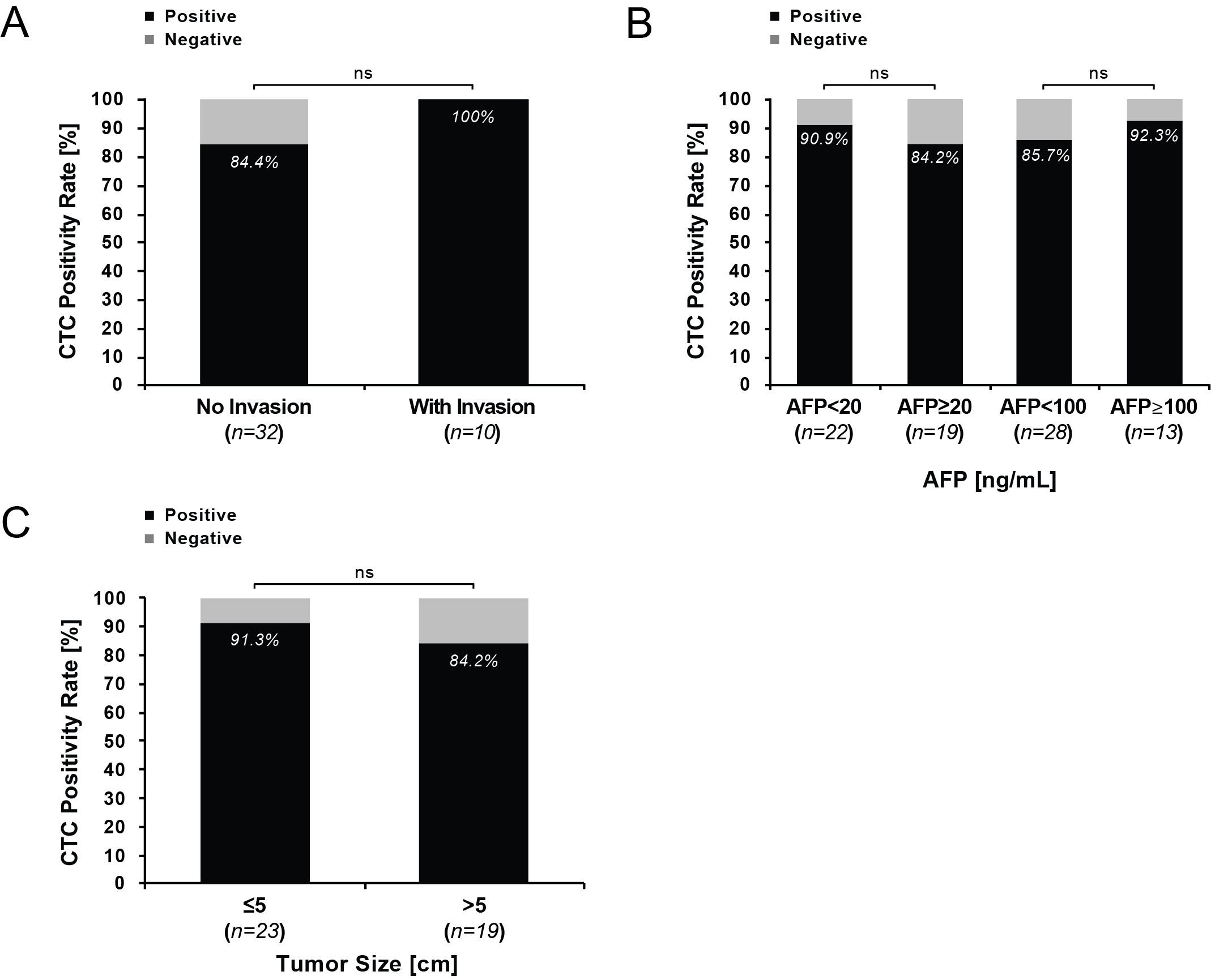


**Supplemental Figure 4. CTC positivity rate and HCC prognostic factors.**

A. CTC positivity rate in HCC patients with or without macrovascular invasion. B. CTC positivity rate in HCC patients with high vs. low serum AFP. Left two columns used cut off AFP value of 20 ng/mL, right two columns used cut off AFP value of 100 ng/mL. C. CTC positivity rate in HCC patients with tumor size smaller or larger than 5 cm.

**
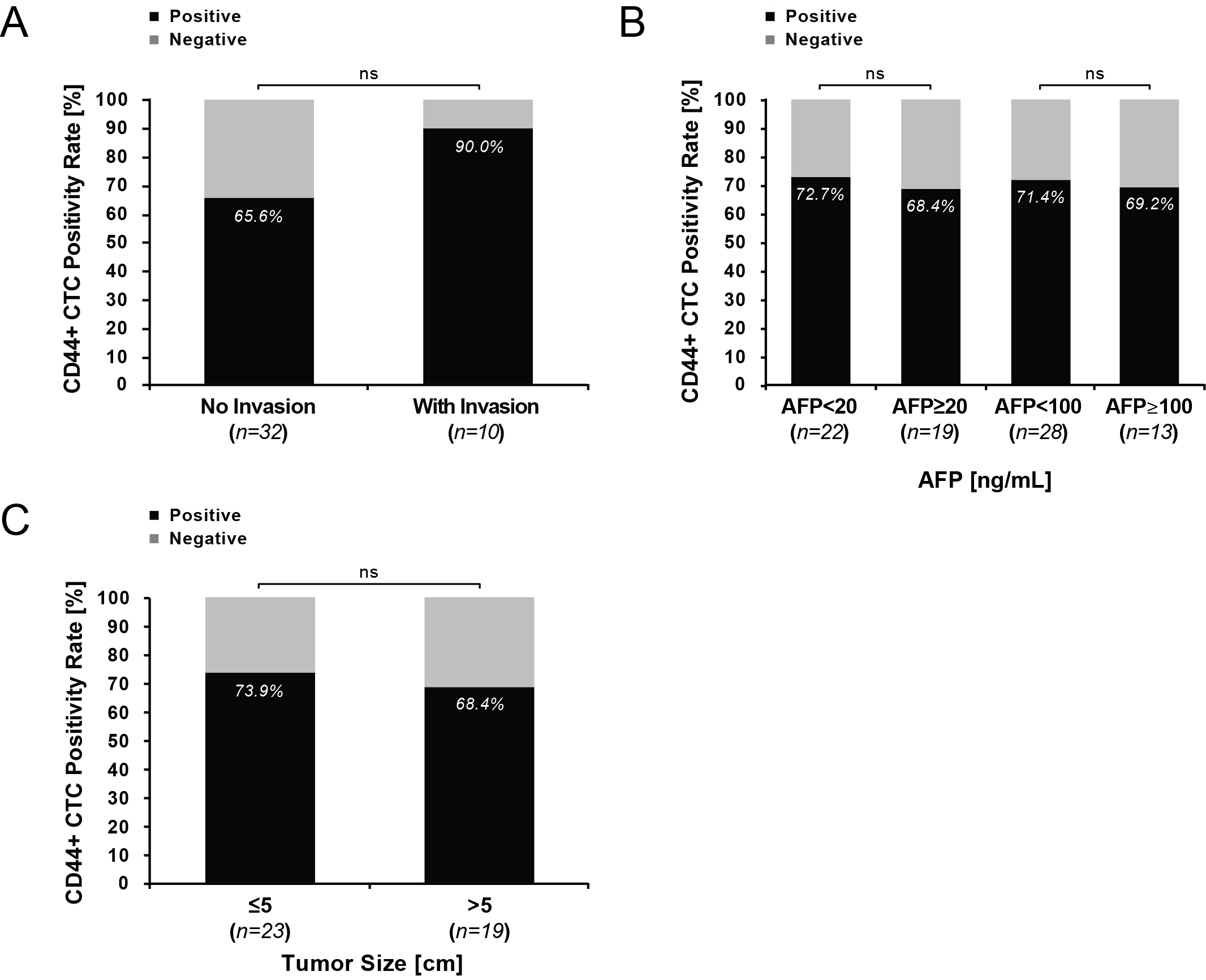
**

**Supplemental Figure 5. CD44 positive CTCs and HCC prognostic factors.**

A. CD44^+^ CTC positivity rate in HCC patients with or without macrovascular invasion. B. CD44^+^ CTC positivity rate in HCC patients with high vs. low serum AFP. Left two columns used cut off AFP value of 20 ng/mL, right two columns used cut off AFP value of 100 ng/mL. C. CD44^+^ CTC positivity rate in HCC patients with tumor size smaller or larger than 5 cm.

**
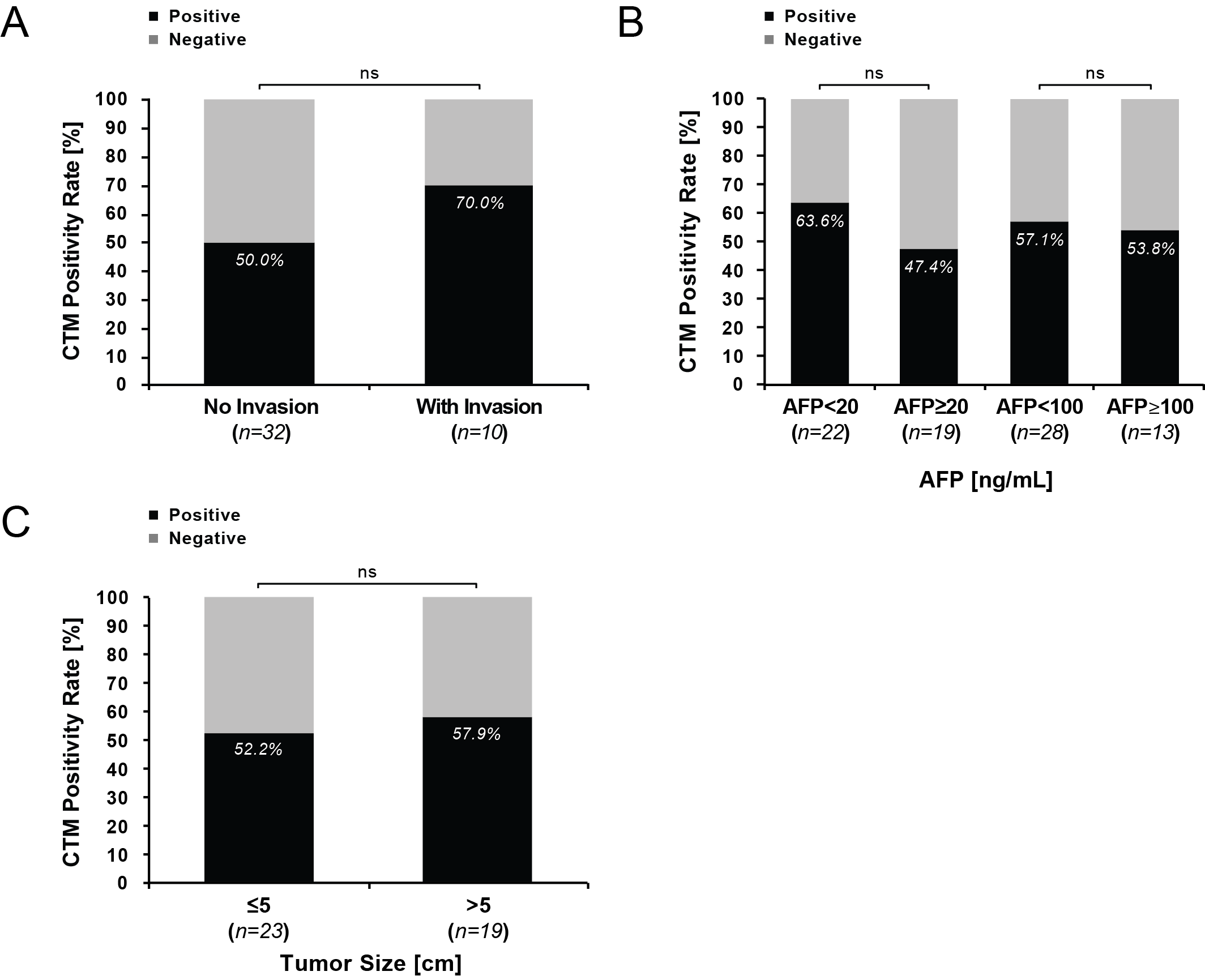
**

**Supplemental Figure 6. CTM and HCC prognostic factors.**

A. CTM positivity rate in HCC patients with or without macrovascular invasion. B. CTM positivity rate in HCC patients with high vs. low serum AFP. Left two columns used cut off AFP value of 20 ng/mL, right two columns used cut off AFP value of 100 ng/mL. C. CTM positivity rate in HCC patients with tumor size smaller or larger than 5 cm.

**Supplemental Table 1.** **Comparison of HCC CTC studies and technologies used for isolation.**

| **First Author** | **CTC Isolation**  **technology** | **CTC**  **Identification** | **Patient**  **Cohort** | **CTC Positivity Rate,**  **Number of CTC / mL** | **Summary** |
| --- | --- | --- | --- | --- | --- |
| **Our Work** | Size-based microfluidic Labyrinth device,  Label free | Three HCC marker + (GPC3, GS, HepPar-1) positive,  CD45 negative | 48 HCC  patients | 88.1%,  3.63 / ml | Positivity of CTC and CD44+ CTC correlate with tumor stages. |
| Schulze, Wege^1^ | Cell Search  Immunoaffinity:  EpCAM-labeled | Cytokeratins positive, CD45 negative | 59 HCC  patients | 30.5%,  0.32 / ml | Correlation with AFP, tumor stage and OS |
| Sun, Xu, Yang^2^ | Cell Search  Immunoaffinity;  EpCAM-labeled | Cytokeratins positive, CD45 negative | 123 HCC patients | 66.67%,  No average number of CTC specified | EpCAM^+^ CTC as independent prognostic indicatior for recurrence. |
| Li, Chen, Zhang^3^ | Immunoaffinity:  ASGPR-labeled | Cytokeratins or HepPar-1 positive,  CD45 negative | 27 HCC  patients | 88.9%,  6.8 / ml | No correlation analyzed for CTC with clinical parameters. |
| Wang^4^ | CanPatrol^TM^  Filtration-based: Membrane filter with a pore size of 8um | RNA in situ hybridization: Epithelial (CK8, 18 and 19, EpCAM), Mesenchymal (Vimentin, Twist), CD45 | 62 HCC  patients | No CTC positivity rate specified,  0.91 / ml | Correlation of mesenchymal CTCs with higher risk of recurrence. |
| Chen, Li^5^ | CanPatrol^TM^  Filtration-based:  Membrane filter with a pore size of 8um | RNA in situ hybridization: Epithelial (CK8, 18 and 19, EpCAM), Mesenchymal (Vimentin, Twist), CD45 | 113 HCC patients before curative treatment and 143 HCC patients after curative treatment | 78.8%  2.24 / ml | No correlation of CTC or EMT before or after curative treatment with recurrence. |
| Liu, Guo, Zhang^6^ | Imaging flow cytometry, based on high Karyoplasmic ratio (HKR) | Flow cytometry to distinguish CD45 negative CTC based on Nuclear-cytoplasmic ratio. | 52 HCC patients | 85.19%  8.4 / ml | Number of CTC associated with microvascular invasion. |
| Kalinich, Bhan^7^ | Microfluidic CTC-iChip. | RNA-based digital CTC scoring | 48 HCC patients | 56%, N/A | No correlation with AFP. CTC score decrease after treatment. |
